# Chemical Characterization and Antimicrobial Activity of Pyrolysis Liquids from Walnut Residue

**DOI:** 10.3390/ijms27094011

**Published:** 2026-04-30

**Authors:** Ibrahim Koc, Erdal Ogun, Fatmagul Geven, Kerim Guney, Faruk Yildiz, Ozkan Kaya

**Affiliations:** 1 Vocational School of Health Services, Mardin Artuklu University, Mardin 47060, Türkiye; 2 Department of Molecular Biology and Genetics, Faculty of Science, Van Yüzüncü Yıl University, Van 65080, Türkiye; 3 Department of Biology, Faculty of Science, Ankara University, Ankara 06100, Türkiye; 4 Department of Forest Engineering, Faculty of Forestry, Kastamonu University, Kastamonu 37150, Türkiye; 5 Department of Biology, Faculty of Arts and Science, Erzincan Binali Yıldırım University, Erzincan 24100, Türkiye; 6 Erzincan Horticultural Research Institute, Republic of Türkiye Ministry of Agriculture and Forestry, Erzincan 24060, Türkiye; 7 Department of Life Sciences, Western Caspian University, Baku 1001, Azerbaijan

**Keywords:** antimicrobial activity, biopesticide, circular economy, pyrolysis liquids, walnut residue, total phenolic

## Abstract

Pyrolysis liquid (PL) derived from biomass pyrolysis exhibits biopesticidal properties and represents a promising value-added product within the sustainable circular economy framework. However, knowledge about the antimicrobial potential of PLs produced from walnut residue at different pyrolysis temperatures remains limited. We investigated the chemical composition and antimicrobial activity of PLs obtained from agricultural walnut residue (*Juglans regia* L.) against selected plant pathogenic bacteria and fungi. PLs were produced at four temperature ranges: 200–300 °C (W-1), 300–400 °C (W-2), 400–500 °C (W-3), and 500–600 °C (W-4). Chemical characterization was performed using Gas chromatography–mass spectrometry (GC-MS), High-performance liquid chromatography (HPLC), and Inductively coupled plasma optical emission spectrometry (ICP-OES), with determination of total phenolic and flavonoid contents. Pyrolysis temperature significantly influenced the chemical profile and bioactive compound content of the PLs, with W-4 showing the highest total phenolic and flavonoid levels. Heavy metal analysis indicated minimal contamination in all samples. Antibacterial activity was observed in stock solutions, whereas diluted applications showed limited effects. The W-4 fraction showed the strongest antibacterial activity and exhibited MIC values of 12.50 µL/mL against *Clavibacter michiganensis* subsp. michiganensis, *Xanthomonas euvesicatoria*, and *Pseudomonas syringae* pv. syringae, and 25.00 µL/mL against *Erwinia amylovora*. Antifungal activity differed markedly across temperature ranges, with W-3 and W-4 displaying superior activity against *Fusarium oxysporum* and *Verticillium dahliae*, achieving complete mycelial growth inhibition at 5%, compared to 10% for W-2 and 20% for W-1. Positive controls confirmed assay validity (ciprofloxacin for antibacterial assays and cycloheximide for antifungal assays), whereas negative controls showed no inhibitory effect. Overall, higher pyrolysis temperatures, particularly 400–600 °C, enhanced the antimicrobial potential of walnut residue-derived PLs, supporting their possible use as bio-based antifungal agents for sustainable crop protection.

## 1. Introduction

Humans depend on plants for numerous essential purposes, including nutrition, clothing, shelter, pharmaceutical production, and industrial raw material supply, making them indispensable for sustaining life on Earth. However, plants suffer qualitative and quantitative losses due to biotic factors such as bacteria, fungi, weeds, nematodes, and arthropods [1,2,3]. Among these factors, phytopathogenic bacteria [4,5] and fungi [3,4,6,7,8] cause particularly severe damage to crop production. Fungal-induced crop losses are re-ported to reach up to 14% annually, and in some cases can even reach 100% [3,8]. This situation poses a significant threat to both agricultural sustainability and global food security [4,8]. Various methods are employed to combat biological factors that harm plants [9], with synthetic chemicals known as pesticides being widely preferred for this purpose [10,11,12,13,14,15,16,17,18,19]. These chemicals are used not only in agricultural fields but also in environmental settings such as forestry, homes, offices, and parks [20,21,22,23,24,25]. Following the Industrial Revolution and especially during the twentieth century, pesticide production and use expanded markedly in parallel with the rapid development of the chemical industry and intensification of modern agriculture [26,27]. The World Health Organization (WHO) classifies pesticides into four categories based on their toxicity: slightly, moderately, highly, and extremely hazardous [28,29]. Nevertheless, pesticides have multifaceted adverse effects on living organisms and ecosystems [1,2,19,30,31,32]. Consequently, the development of biopesticides is gaining increasing importance to reduce pollution caused by chemical pesticides and limit their negative impacts on non-target organisms [1,33,34]. Biopesticides derived from plant-based or microbial compounds are emerging as environmentally friendly “green solutions” [2]. Pyrolysis liquid (PL) obtained through biomass pyrolysis has been identified in the literature as one of the products exhibiting biopesticidal properties [31,34,35,36,37,38,39,40,41,42,43,44,45,46]. This product is also referred to in the literature by various names such as wood vinegar, pyrolysis oil, pyrolytic liquor, or pyrolytic tar [47,48]. PL is a complex mixture with acidic characteristics, high water content, and composition of both organic and inorganic components [16,34,46,49,50,51,52,53,54].

Recent studies have demonstrated the diverse chemical composition and antimicrobial potential of biomass-derived PLs. Yin et al. [43] identified over 70 oxygenated organic compounds in PL obtained from *Coptis chinensis* Franch. at 400 °C. Koç [34] detected 53 components via Gas chromatography–mass spectrometry (GC-MS) and 12 components via chromatography–tandem mass spectrometry (LC-MS/MS) in PL from green walnut husks (*J. regia* L.), and determined total phenolic and flavonoid contents. Antimicrobial tests showed concentration-dependent activity, with maximum antifungal efficacy at 25% (*v*/*v*). Koç [46] identified 146 components in PL from oak residues (*Quercus* sp.) pyrolyzed at 100–550 °C, with concentrations above 2% (*v*/*v*) achieving complete fungal growth inhibition. Öğün and Koç [41] examined PLs from hazelnut shells and broiler litter against phytopathogenic bacteria, finding resistance to broiler litter PL but susceptibility to hazelnut shell PL. These exemplary studies demonstrate that biomass contains numerous bioactive components and that these components can be effectively extracted through pyrolysis. Therefore, converting biomass into value-added products such as biopesticides for sustainable agriculture, food, and environmental applications forms the foundation of the circular economy approach [46]. The structure and efficacy of PL are directly related to parameters such as reactor type, feedstock characteristics, and pyrolysis temperature [34,46,55,56,57]. A review of the literature reveals that the effects of PL obtained from agricultural walnut residue (*J. regia*, Juglandaceae) against plant pathogenic fungi and bacteria have not yet been investigated. Walnut is one of the important species widely cultivated worldwide [58,59]. As of 2022, the global walnut cultivation area is 1.2 million hectares, with Türkiye ranking second in world production with 166 thousand hectares [60]. For this reason, determining the chemical profile of PL produced from walnut waste and investigating its biological activity against plant pathogenic organisms is of both economic and environmental importance.

In this perspective, the objectives of the study are as follows: (I) to determine the chemical composition of PLs obtained from agricultural walnut residue (*J. regia*) at four different temperature ranges using multiple analytical methods; (II) to assess the antimicrobial performance of these PLs against selected plant pathogenic bacteria and fungi, with a focus on concentration-dependent efficacy during in vitro testing; (III) to analyze the relationship between pyrolysis temperature and bioactive compound formation, with the goal of identifying optimal production parameters for biopesticide development; and (IV) to evaluate the potential of walnut residue-derived PLs as value-added products in sustainable agriculture and circular economy applications, providing valuable insights for future research aimed at developing bio-based crop protection products. By achieving these objectives, the study seeks to contribute essential knowledge for valorizing agricultural walnut waste into environmentally friendly biopesticides, thus supporting sustainable pest management practices in the agricultural sector.

## 2. Results

### 2.1. Characterization of PLs

#### 2.1.1. Total Phenolic and Total Flavonoid Levels

The rich phenolic content of PLs was calculated as gallic acid equivalents, while the flavonoid content was expressed as quercetin equivalents. The standard gallic acid calibration curve formula was Y = 0.8234x − 0.0058 (R^2^ = 0.9953), and the quercetin equivalent formula was Y = 2.2059x − 0.0408 (R^2^ = 0.9931). As shown in Table 1, both total phenolic and total flavonoid contents increased progressively with rising pyrolysis temperature. Total phenolic content ranged from 65.53 ± 8.00 mg GAE/mL in W-1 to 321.18 ± 10.50 mg GAE/mL in W-4, representing nearly a five-fold increase. Similarly, total flavonoid content increased from 16.37 ± 1.89 mg QE/mL in W-1 to 77.11 ± 2.36 mg QE/mL in W-4. The intermediate temperature ranges W-2 (300–400 °C) and W-3 (400–500 °C) showed total phenolic contents of 146.05 ± 5.50 and 233.62 ± 6.00 mg GAE/mL, respectively, with corresponding flavonoid contents of 32.48 ± 1.41 and 55.90 ± 3.77 mg QE/mL.

#### 2.1.2. Volatile Aromatic Compounds Determined by GC-MS (SPME)

GC–MS analysis revealed clear temperature-dependent differences in the chemical composition of PLs obtained from walnut residue (Table 2). In W-1 (200–300 °C), furfural (14.78%) and hydrazine, 1,1-dimethyl- (12.18%) were the dominant compounds, together with acetic acid, propanedioic acid, and several fatty acids. In W-2 (300–400 °C), L-alanine (14.98%) and 1,2-propanediamine (9.74%) became prominent, whereas furfural decreased compared with W-1. In W-3 (400–500 °C), the main compounds were hydrazine, 1,1-dimethyl- (12.20%) and 1-propanol, 2-amino- (10.64%), accompanied by nitrogen-containing derivatives and moderate levels of furfural. W-4 (500–600 °C) exhibited the highest chemical diversity, with propanoic acid (11.58%) and 2(3H)-furanone, dihydro- (8.86%) as the dominant constituents, together with guaiacol, pyridine, formic acid, and several lactone derivatives. Overall, increasing pyrolysis temperature was associated with a marked decline in furfural content and a progressive enrichment of organic acids, phenolic/aromatic compounds, and lactones, particularly in the W-4 fraction.

The total phenolic and total flavonoid contents of the PLs varied significantly according to pyrolysis temperature (Table 3). Both parameters generally increased with increasing temperature, and the highest values were recorded in the W-4 fraction. Intermediate levels were observed in W-2 and W-3, whereas W-1 exhibited the lowest contents. These findings indicate that higher pyrolysis temperatures promoted the formation or release of phenolic and flavonoid-derived constituents in walnut residue-derived PLs.

It should be noted that GC–MS identification was performed based on mass spectral library matching, and some nitrogen-containing compounds may represent structurally related derivatives or co-eluting species inherent to complex pyrolysis mixtures.

#### 2.1.3. Phenolic Profile

HPLC analysis revealed distinct temperature-related differences in the phenolic acid composition of the PLs (Table 4). Gallic acid was the predominant phenolic compound in all samples and reached its highest level in W-1, followed by a gradual decrease at higher temperatures. In contrast, caffeic acid increased progressively with pyrolysis temperature and showed its maximum concentration in W-4. Vanillic acid was most abundant in W-3, whereas *p*-coumaric acid remained the least abundant phenolic acid in all fractions, although its concentration increased with temperature. Overall, the results indicate that pyrolysis temperature influenced both the relative distribution and abundance of individual phenolic acids in walnut residue-derived PLs.

#### 2.1.4. Elemental Analysis by ICP-OES

ICP-OES analysis demonstrated distinct mineral composition patterns among the pyrolysis liquid samples (Table 5). Heavy metals, including Cu, Mn, Sn, Cd, Ni, Pb, Hg, and Cr were below the detection limits in all fractions. Among the detected elements, calcium was present at the highest concentrations, with the maximum level observed in W-2. Iron and zinc were also abundant, particularly in W-2, W-3, and W-4. Magnesium showed moderate variation among fractions and reached its highest concentration in W-4. Potassium and sodium levels were relatively stable across samples, with only minor differences. These findings indicate that walnut residue-derived PLs contain appreciable mineral constituents while remaining free of detectable toxic heavy metal contamination under the present experimental conditions.

### 2.2. Antimicrobial Activities

#### 2.2.1. Antibacterial Activities

**Figure 1:**
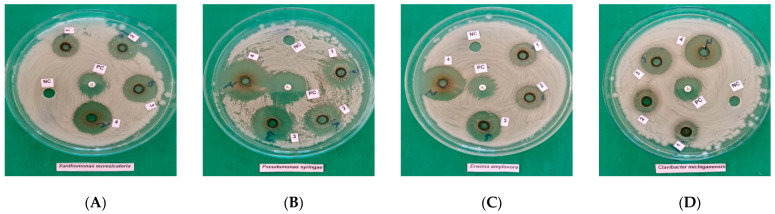
Representative agar well diffusion plates illustrating antibacterial inhibition zones produced by stock pyrolysis liquids (W-1 to W-4). (**A**): W-1 (200–300 °C); (**B**): W-2 (300–400 °C); (**C**): W-3 (400–500 °C); (**D**): W-4 (500–600 °C); PC: positive control (ciprofloxacin); NC: negative control.

To first confirm the intrinsic antibacterial potential of walnut-derived PLs independently of dilution effects, stock PLs (100% *v*/*v*) were evaluated against phytopathogenic bacteria using the agar well diffusion method. Under these non-diluted conditions, all stock fractions (W-1–W-4) produced clear and measurable inhibition zones against all tested bacterial species (Table 6, Figure 1). The inhibition zone diameters increased consistently with rising pyrolysis temperature, and the highest-temperature fraction (W-4) exhibited the strongest antibacterial activity across all pathogens. One-way analysis of variance (ANOVA) revealed statistically significant differences among PL fractions for each bacterial species tested (*p* < 0.05), confirming a clear temperature-dependent antibacterial trend. These results demonstrate that the antibacterial efficacy of pyrolysis liquids becomes pronounced at full-strength application and is strongly influenced by pyrolysis temperature.

Representative agar well diffusion images corresponding to the confirmatory antibacterial assays performed with stock PLs are shown in Figure 1, visually supporting the quantitative inhibition zone data presented in Table 6.

**Figure 2:**
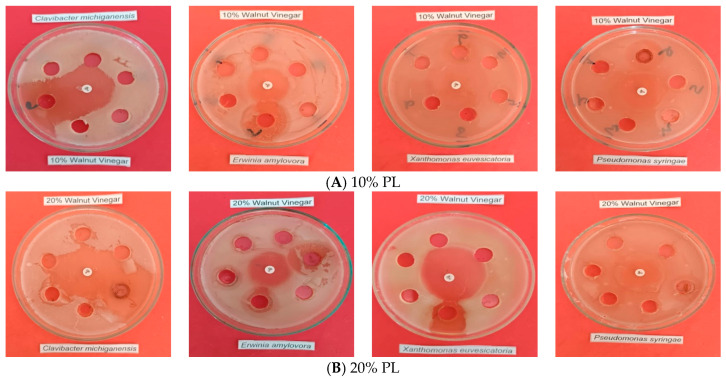
Inhibition zone diameters of pyrolysis liquids (W-1, W-2, W-3, W-4) against phytopatho-genic bacteria at (**A**) 10% and (**B**) 20% (*v*/*v*) concentrations, with ciprofloxacin used as a positive control. Note: Numbers 1 to 6 represent W-1, W-2, W-3, W-4, negative control (TSB), and stock solution (positive control, 100% PL), respectively.

Following confirmation of antibacterial activity under non-diluted conditions, the effects of dilution were subsequently evaluated to assess antibacterial performance at practical application levels. Antibacterial screening of diluted pyrolysis liquids at 10% and 20% (*v*/*v*) concentrations revealed markedly reduced and concentration-dependent inhibitory effects against all tested phytopathogenic bacteria (Table 7, Figure 2). At the 10% PL (*v*/*v*) concentration, none of the diluted pyrolysis liquid fractions (W-1–W-4) produced detectable inhibition zones against *Clavibacter michiganensis* subsp. *michiganensis*, *Erwinia amylovora*, *Pseudomonas syringae* pv. *syringae*, or *Xanthomonas euvesicatoria*, indicating complete loss of antibacterial activity at this dilution level. At the 20% PL (*v*/*v*) concentration, antibacterial activity remained largely absent for most diluted treatments. However, a measurable inhibition zone was observed exclusively for the W-4 fraction against *E. amylovora* (15 mm), while all other diluted PL applications showed no inhibitory effect against the remaining pathogens. These findings demonstrate that dilution substantially diminishes the antibacterial efficacy of walnut-derived PLs, and that detectable antibacterial activity at reduced concentrations is limited to specific pathogen–fraction combinations, with the highest-temperature fraction (W-4) exhibiting comparatively greater resilience to dilution.

The negative control (TSB medium) produced no inhibition zones against any bacterial pathogen, whereas the positive control (ciprofloxacin, 5 µg) exhibited broad-spectrum antibacterial activity at both concentrations. In the 10% PL (*v*/*v*) medium, ciprofloxacin showed the highest activity against *P. syringae* pv. *syringae* (30 mm), followed by *C. michi-ganensis* subsp. *michiganensis* and *E. amylovora* (25 mm), with the lowest activity observed against *X. euvesicatoria* (20 mm). In the 20% PL (*v*/*v*) medium, ciprofloxacin exhibited enhanced inhibition of *C. michiganensis* subsp. *michiganensis* (35 mm) and *P. syringae* pv. *sy-ringae* (30 mm), maintained activity against *E. amylovora* (25 mm), but showed reduced ef-ficacy against *X. euvesicatoria* (10 mm).

**Minimum inhibitory concentration (MIC) determination.**

**Figure 3:**
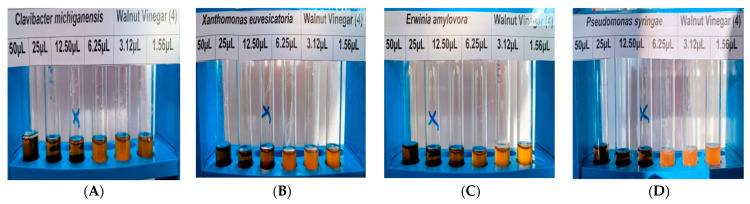
Minimum inhibitory concentration (MIC) assay illustrating concentration-dependent growth inhibition of phytopathogenic bacteria by the W-4 pyrolysis liquid fraction (500–600 °C). (**A**): *Clavibacter michiganensis* subsp. *michiganensis*, (**B**): *Xanthomonas euvesicatoria*, (**C**): *Pseudomonas syringae* pv. *syringae*, (**D**): *Erwinia amylovora*.

Minimum inhibitory concentration (MIC) assays were conducted in triplicate for all four phytopathogenic bacteria to quantitatively confirm the antibacterial efficacy of walnut-derived PLs. Based on agar well diffusion results, the highest-temperature fraction (W-4; 500–600 °C), which consistently exhibited the strongest antibacterial activity, was selected for MIC determination. The MIC values of W-4 were determined as 12.50 µL/mL for *C. michiganensis* subsp. *michiganensis*, *X. euvesicatoria*, and *P. syringae* pv. *syringae*, whereas a higher MIC value of 25.00 µL/mL was observed for *E. amylovora*. These results demonstrate a clear pathogen-dependent susceptibility pattern and confirm that W-4 exerts strong concentration-dependent antibacterial effects, with *E. amylovora* exhibiting comparatively higher tolerance (Table 8, Figure 3).

#### 2.2.2. Antifungal Activities

**Figure 4:**
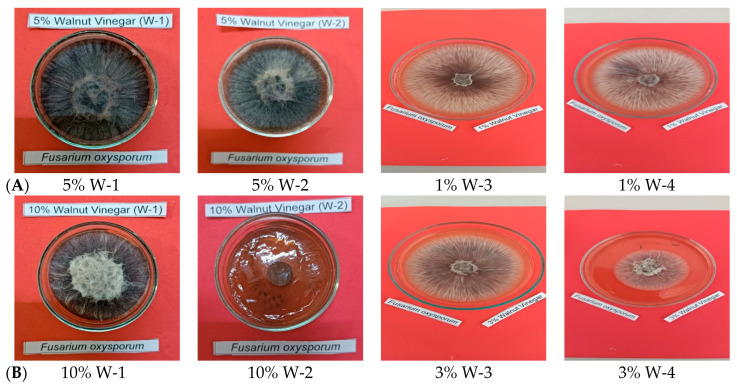

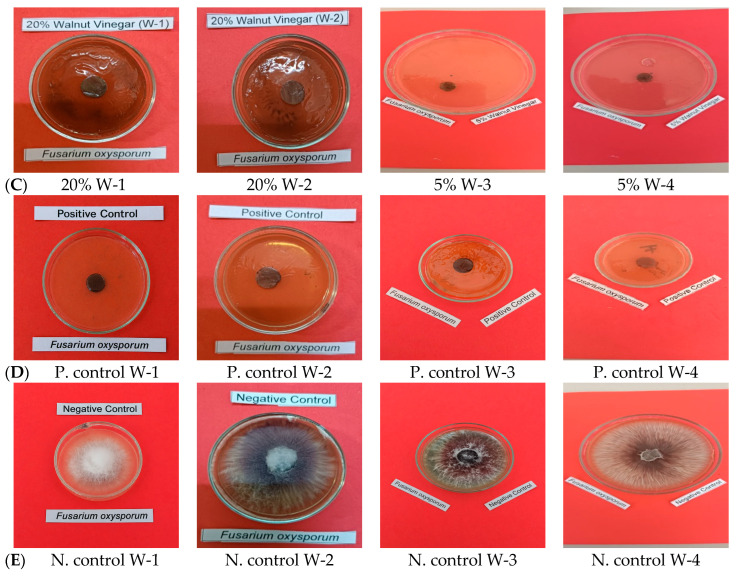
Mycelial growth inhibition of *F. oxysporum* by pyrolysis liquids (W-1, W-2, W-3, W-4) at 5%, 10%, and 20% (*v*/*v*) concentrations on MEA medium with positive and negative controls. (**A**) Effect of W-1 pyrolysis liquid at 5%, 10%, and 20% (*v*/*v*) concentrations on mycelial growth of *F. oxysporum*. (**B**) Effect of W-2 pyrolysis liquid at 5%, 10%, and 20% (*v*/*v*) concentrations on mycelial growth of *F. oxysporum*. (**C**) Effect of W-3 pyrolysis liquid at 1%, 3%, and 5% (*v*/*v*) concentrations on mycelial growth of *F. oxysporum*. (**D**) Effect of W-4 pyrolysis liquid at 1%, 3%, and 5% (*v*/*v*) concentrations on mycelial growth of *F. oxysporum*. (**E**) Positive control (cycloheximide) and negative control showing inhibited and normal mycelial growth of *F. oxysporum*, respectively.

Antifungal screening of PLs against *F. oxysporum* revealed concentration-dependent inhibitory effects on mycelial growth (Figure 4). Malt Extract Agar medium supplemented with W-1 at varying concentrations demonstrated fungal growth at 5% (*v*/*v*) and 10% (*v*/*v*), while complete growth inhibition was observed at 20% (*v*/*v*) concentration. Medium containing W-2 exhibited fungal growth at 5% (*v*/*v*) and 20% (*v*/*v*) concentrations, whereas 10% (*v*/*v*) concentration completely suppressed mycelial development. Media supplemented with W-3 and W-4 showed similar antifungal patterns, with fungal growth occurring at 1% (*v*/*v*) and 3% (*v*/*v*) concentrations, while 5% (*v*/*v*) concentration resulted in complete growth inhibition for both samples. The negative controls (W-1, W-2, W-3, W-4) demonstrated uninhibited mycelial growth of *F. oxysporum*, confirming the absence of contamination and viability of the fungal inoculum. Conversely, positive controls (W-1, W-2, W-3, W-4) exhibited complete absence of fungal growth, validating the efficacy of cycloheximide as an antifungal reference compound. Our results indicated that pyrolysis liquids possess fungistatic properties that are directly proportional to concentration, with higher concentrations achieving complete inhibition of *F. oxysporum* mycelial growth across all temperature-variant samples.

**Figure 5:**
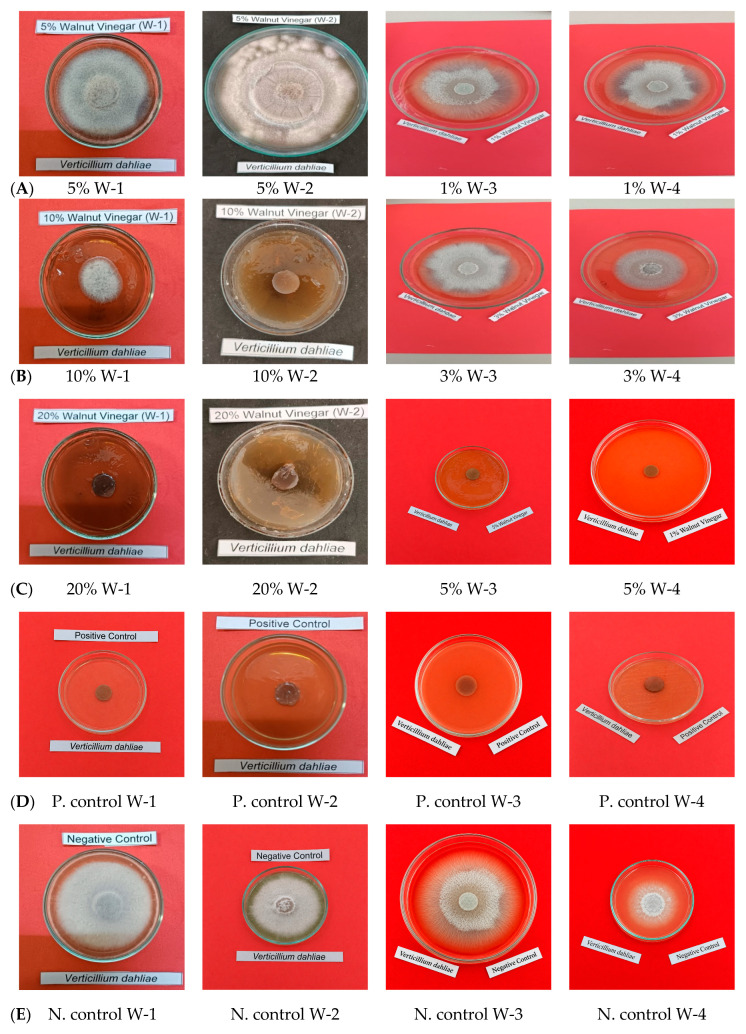
Mycelial growth inhibition of *Verticillium dahliae* by pyrolysis liquids (W-1, W-2, W-3, W-4) at 5%, 10%, and 20% (*v*/*v*) concentrations on MEA medium with positive and negative controls. (**A**) Effect of W-1 pyrolysis liquid at 5%, 10%, and 20% (*v*/*v*) concentrations on mycelial growth of *V. dahliae*. (**B**) Effect of W-2 pyrolysis liquid at 5%, 10%, and 20% (*v*/*v*) concentrations on mycelial growth of *V. dahliae*. (**C**) Effect of W-3 pyrolysis liquid at 1%, 3%, and 5% (*v*/*v*) concentrations on mycelial growth of *V. dahliae*. (**D**) Effect of W-4 pyrolysis liquid at 1%, 3%, and 5% (*v*/*v*) concentrations on mycelial growth of *V. dahliae*. (**E**) Positive control (cycloheximide) and negative control showing inhibited and normal mycelial growth of *V. dahliae*, respectively.

Antifungal screening of PLs against *V. dahliae* demonstrated concentration-dependent inhibitory effects on mycelial development (Figure 5). MEA medium supplemented with W-1 exhibited fungal growth at all concentrations except 20% (*v*/*v*), where complete growth inhibition was achieved. Medium containing W-2 demonstrated mycelial growth at 5% (*v*/*v*) and 20% (*v*/*v*) concentrations, while 10% (*v*/*v*) concentration completely suppressed fungal development. Media supplemented with W-3 and W-4 showed similar antifungal patterns, with fungal growth occurring at 1% (*v*/*v*) and 3% (*v*/*v*) concentrations, whereas 5% (*v*/*v*) concentration resulted in complete mycelial growth inhibition for both samples. The negative controls (W-1, W-2, W-3, W-4) demonstrated uninhibited mycelial growth of *V. dahliae*, confirming the viability of the fungal inoculum and absence of contamination. Conversely, positive controls (W-1, W-2, W-3, W-4) exhibited complete absence of fungal growth, validating the efficacy of the antifungal reference compound.

Walnut-derived PLs showed clear temperature-dependent antifungal activity against both tested pathogens (Table 9). Complete mycelial growth inhibition of *F. oxysporum* and *V. dahliae* was achieved at 20% for W-1, 10% for W-2, and 5% for the higher-temperature fractions W-3 and W-4, indicating greater antifungal potency in PLs produced at elevated pyrolysis temperatures (Table 9).

## 3. Discussion

### 3.1. Chemical Characterization and Compositional Diversity of Pyrolysis Liquids

The comprehensive GC–MS analysis revealed distinct temperature-dependent chemical profiles in walnut-derived PLs, demonstrating significant variations in compound distribution across the four temperature ranges examined (Table 2 and Table 3). These compositional shifts reflect the complex thermal decomposition pathways of walnut biomass components during pyrolysis. In the organic acids and fatty acids category, the progressive increase in propanoic acid concentration from 4.08% (W-1) to 11.58% (W-4) indicates intensified thermal cracking of complex macromolecules at elevated temperatures (Table 3). This trend aligns with observations by Başar et al. [61], who documented similar temperature-dependent shifts in organic acid composition during biomass pyrolysis. The presence of acetic acid (6.39%) and propanedioic acid (7.01%) exclusively in W-1, along with long-chain fatty acids such as 9,12-octadecadienoic acid (1.75%) and 9-octadecenoic acid (1.81%), suggests that lower pyrolysis temperatures favor the preservation of thermally labile compounds (Table 1). The appearance of formic acid at higher temperatures (2.21% in W-2, increasing to 3.83% in W-4) reflects the enhanced decarboxylation and oxidation reactions occurring under more severe thermal conditions, consistent with mechanisms described by El-Fawy et al. [54]. The furans and lactones group exhibited a remarkable temperature-dependent transformation pattern. Furfural, derived primarily from hemicellulose degradation, decreased progressively from 14.78% in W-1 to 1.36% in W-4 (Table 3), indicating thermal decomposition of this compound at elevated temperatures. This observation corroborates findings by Mela et al. [37] and Feng et al. [53], who identified furfural as a primary product of hemicellulose pyrolysis at moderate temperatures. The emergence of various lactone compounds exclusively in W-4, particularly 2(3H)-furanone, dihydro- at 8.86%, suggests that higher temperatures promote cyclization and rearrangement reactions leading to thermally stable heterocyclic structures (Table 1). The presence of γ-butyrolactone in W-1 (1.94%) and W-2 (2.68%) further demonstrates the temperature-specific formation of oxygenated heterocycles, as reported by Liu et al. [62] in their investigation of lignocellulosic pyrolysis products.

Phenolic and aromatic compounds represent a critical chemical class with significant implications for antimicrobial activity. The detection of guaiacol in both W-1 (1.54%) and W-4 (3.04%) indicates lignin depolymerization across temperature ranges (Table 3). The exclusive presence of benzenesulfonic acid, 4-hydroxy- (4.01%) in W-4 suggests that elevated temperatures facilitate the formation of sulfonated aromatic compounds through complex thermal reactions (Table 2). Pyridine concentration increased progressively from 1.02% (W-1) to 3.63% (W-4), reflecting nitrogen incorporation into aromatic ring systems at higher temperatures, consistent with observations by Yang et al. [63]. These methoxylated and hydroxylated phenols likely originate from lignin degradation, as lignin-derived phenolic structures undergo demethoxylation and recombination reactions at elevated temperatures, as described by Koç [46]. The nitrogen-containing compounds exhibited the most complex temperature-dependent behavior among all chemical classes examined. L-alanine reached its maximum concentration of 14.98% in W-2, accompanied by 1,2-propanediamine at 9.74% (Table 3), suggesting that intermediate temperatures (300–400 °C) provide optimal conditions for amino acid liberation from protein-rich walnut tis-sues. The alternating presence of hydrazine, 1,1-dimethyl- in W-1 (12.18%) and W-3 (12.20%), but not in W-2 and W-4, indicates temperature-specific formation mechanisms involving nitrogen-containing precursors (Table 2). The appearance of various nitro com-pounds, including methane, trinitro- (9.55%) in W-1 and methane, nitro- (7.73%) in W-3, reflects the oxidative thermal transformation of nitrogen species under different temperature regimes. These findings are consistent with the thermal decomposition patterns described by Li et al. [64] and Ouattara et al. [65] for nitrogen-rich biomass feedstocks. The alcohols and polyols group demonstrated distinct temperature preferences. The substantial concentrations of 1-propanol-O-D (6.49%) and 2-propanol (5.04%) in W-2, along with 1,2,3-propanetriol (3.88%), indicate that moderate temperatures favor the formation of simple alcohols and polyols through cellulose and hemicellulose degradation (Table 3). The exclusive detection of 1-propanol, 2-amino- at 10.64% in W-3 suggests that this tem-perature range promotes the formation of amino alcohols through combined dehydration and amination reactions (Table 3). These alcohol distribution patterns align with the thermal decomposition pathways reported by Mela et al. [37] for carbohydrate-rich bio-mass materials. The chemical diversity observed in walnut PLs, with W-4 containing 25 major compounds and exhibiting the highest compositional complexity, exceeded findings reported by Li et al. [64] and Ouattara et al. [65], though remained lower than the 146 components identified by Koç [46] in oak-derived pyrolysis liquids. This indicates feedstock-specific variations in thermal decomposition products. The temperature-dependent chemical transformations documented in Table 2 and Table 3 demonstrate that pyrolysis conditions can be optimized to produce PLs enriched in specific bioactive compounds. The progressive increase in total phenolic content from 65.53 ± 8.00 mg GAE/mL (W-1) to 321.18 ± 10.50 mg GAE/mL (W-4), representing a nearly five-fold enhancement, underscores the critical role of thermal processing conditions in phenolic liberation and concentration, as observed by Liu et al. [62] and Yang et al. [63] in comparable lignocellulosic feedstocks.

### 3.2. Antimicrobial Properties and Pathogen-Specific Activity Patterns

The MIC results presented in this study further corroborate the antibacterial screening data, demonstrating that the W-4 fraction exhibits low MIC values (12.50–25.00 µL/mL) against major phytopathogenic bacteria and confirming its comparatively higher antibacterial potency among the tested PLs. In our findings, the antibacterial screening revealed intriguing pathogen-specific and concentration-dependent inhibitory patterns, with undiluted PLs (100% PL) demonstrating selective efficacy against distinct phytopathogenic bacteria. The selective antibacterial activity patterns observed under non-diluted conditions, including the exclusive activity of the W-4 fraction against *C. michiganensis* subsp. *michiganensis*, the preferential inhibition of *E. amylovora* by W-3, the specificity of W-2 toward *X. euvesicatoria*, and the targeted activity of W-1 against *P. syringae* pv. *syringae*, suggest that pyrolysis temperature-dependent chemical composition directly influences the antimicrobial spectrum and potency of walnut-derived PLs. This pathogen-specific activity pattern partially aligns with observations by Öğün and Koç [41] and Feng et al. [53], who reported variable antibacterial efficacy depending on pyrolysis conditions and bacterial strain characteristics. The general absence of antibacterial activity in diluted samples (10% and 20% PL concentrations) contrasts with findings by Xue et al. [66] and Chang et al. [67], who documented sustained antimicrobial activity at comparable dilutions. This discrepancy likely reflects threshold concentration requirements for bioactive compounds, wherein dilution reduces phenolic and organic acid concentrations below minimum inhibitory levels. The differential bacterial susceptibility observed across pyrolysis liquid fractions may stem from variations in cell wall structure, membrane composition, and intrinsic resistance mechanisms among Gram-negative phytopathogens, as discussed by Koç [46]. Furthermore, the complex chemical composition of PLs, comprising phenolics, organic acids, and furan derivatives, suggests multi-target antimicrobial mechanisms involving membrane disruption, enzyme inhibition, and metabolic interference.

In stark contrast to the modest antibacterial effects, the antifungal screening demonstrated robust and consistent activity against both *F. oxysporum* and *V. dahliae*, with clear concentration-response relationships. Complete mycelial growth inhibition occurred at 20% concentration for W-1, 10% for W-2, and 5% for both W-3 and W-4, indicating enhanced antifungal potency at higher pyrolysis temperatures. This inverse relationship between required inhibitory concentration and pyrolysis temperature correlates directly with increased phenolic content and chemical complexity in higher-temperature fractions. These findings align closely with observations by Koç et al. [38,39], Guo et al. [40], Yin et al. [43], and Koç [34,46], all of whom reported substantial antifungal activity in bio-mass-derived PLs. The superior antifungal efficacy compared to antibacterial activity may be attributed to fundamental differences between prokaryotic and eukaryotic cell structures. Fungal cell membranes contain ergosterol as the primary sterol component, rendering them particularly susceptible to phenolic compounds that disrupt membrane fluidity and integrity. Additionally, the lower pH of PLs, resulting from high concentrations of organic acids (particularly acetic acid at 8.59–12.71%), creates an unfavorable environment for fungal growth while simultaneously enhancing the antimicrobial efficacy of weak organic acids through increased membrane permeability in un-dissociated forms. As proposed by Koç [46], the antifungal mechanism likely involves synergistic interactions between phenolic compounds that disrupt cellular membranes and organic acids that interfere with intracellular pH homeostasis and enzymatic functions. The observed concentration-dependent antifungal activity demonstrates practical applicability potential, as the effective concentrations (5–20%) represent feasible dilution ranges for agricultural deployment. The complete growth inhibition achieved at these concentrations indicates pronounced antifungal efficacy under the tested in vitro conditions, with clear suppression of mycelial growth relative to the untreated control. This distinction holds significant practical importance for disease management applications, as fungistatic effects provide more durable pathogen control compared to reversible growth inhibition. A notable outcome of this study is the clear distinction between anti-bacterial and antifungal efficacy of walnut-derived PLs. While antibacterial activity was evident primarily at full-strength application and showed pathogen-dependent sensitivity patterns, antifungal activity was consistently strong even at lower concentrations, particularly for the higher-temperature fractions. This differential bioactivity can be attributed to fundamental structural and physiological differences between bacterial and fungal cells. Fungal membranes, characterized by the presence of ergo sterol and a higher susceptibility to phenolic compounds and organic acids, are more prone to membrane disruption and intracellular acidification. In contrast, bacterial cell envelopes, especially those of Gram-negative phytopathogens, possess outer membrane barriers that can reduce the penetration and effectiveness of complex bioactive mixtures. The low MIC values obtained for the W-4 fraction against multiple bacterial species, together with its superior antifungal performance, highlight the pivotal role of pyrolysis temperature in shaping both chemical composition and biological selectivity of the resulting liquids.

### 3.3. Implications for Sustainable Valorization and Circular Economy Integration

The comprehensive characterization of walnut-derived pyrolysis liquids reveals substantial potential for valorization within circular economy frameworks, transforming agricultural residues into value-added products with demonstrated antimicrobial properties. The abundant availability of walnut (*J. regia*) cultivation residues globally, and particularly in Türkiye [58,59,61], presents significant opportunities for sustainable biomass utilization through slow pyrolysis processing. The thermal conversion of these agricultural wastes into bioactive PLs addresses multiple sustainability objectives: waste reduction, renewable resource development, and production of bio-based antimicrobial agents. The demonstrated antimicrobial properties, particularly the robust antifungal activity against economically important phytopathogens such as *F. oxysporum* and *V. dahliae*, observed at practical dilution ranges (5–20%), position these PLs as promising candidates for bio-based plant protection products. The severe economic losses caused by these soil borne pathogens in numerous crop systems, combined with increasing restrictions on synthetic fungicides due to environmental and health concerns, create urgent demand for effective biological alternatives. Commercial biopesticides based on plant extracts, essential oils, and microbial agents have shown varying efficacy against these pathogens in previous studies [68,69,70,71]; therefore, direct benchmarking of walnut-derived PLs under comparable conditions will be necessary to clarify their practical competitiveness. The phenolic-rich composition of walnut pyrolysis liquids, particularly at higher pyrolysis temperatures, provides multiple bioactive mechanisms that may reduce the likelihood of pathogen resistance development compared to single-target synthetic compounds.

Beyond direct antimicrobial applications, the complex chemical composition of PLs suggests additional potential uses in agriculture, including soil amendment for disease suppression [42], seed treatment formulations [72], and integrated pest management systems. The presence of beneficial mineral elements alongside bioactive organic compounds enhances the potential for multi-functional agricultural products that simultaneously provide plant nutrition and pathogen control. Furthermore, the absence of toxic heavy metals below detection limits addresses critical safety requirements for agricultural product development. The integration of pyrolysis liquid production into walnut cultivation systems represents a promising circular economy model, wherein agricultural residues previously considered waste materials are transformed into valuable products. This approach aligns with broader sustainability transitions in agricultural systems, as emphasized by Cândido et al. [73], Sivaram et al. [45], and Derbali et al. [74], who advocate for biomass valorization strategies that enhance resource efficiency while generating economic value. In addition, the pyrolysis process may generate co-products such as biochar and combustible gases, which could further improve overall process economics through energy recovery and soil improvement applications.

The observed temperature-dependent variations in chemical composition and antimicrobial activity provide valuable guidance for optimizing pyrolysis conditions based on intended applications. For maximum antifungal potency and phenolic concentration, the 500–600 °C temperature range (W-4) appears most advantageous. However, intermediate temperatures may offer advantages for specific applications requiring particular chemical profiles or balancing bioactivity with production costs. Future research should focus on formulation optimization to stabilize bioactive constituents, particularly phenolics, from the W-4 fraction; clarification of structure–activity relationships involving compounds such as propanoic acid and gallic acid; validation of efficacy against *F. oxysporum* and *V. dahliae* under greenhouse and field conditions; assessment of environmental fate and non-target safety; and comparison of production costs with currently available commercial biopesticides. The successful development and deployment of walnut pyrolysis liquid-based products could establish a replicable model for agricultural residue valorization applicable to numerous other crop systems. As articulated by Ayhan and Ayaz [75] and Ouattara et al. [65], the transition toward bio-based economies requires innovative approaches that simultaneously address waste management challenges, reduce dependence on synthetic chemicals, and create economic opportunities in rural communities. The present findings contribute to this broader objective by demonstrating the technical feasibility and biological efficacy of pyrolysis liquid production from abundant agricultural residues, thereby supporting the development of more sustainable and circular agricultural production systems.

## 4. Materials and Methods

### 4.1. Collection Site and Characteristics of Walnut Residue

The agricultural walnut residue (*J. regia*) used in this study was collected on 16 November 2024, from an orchard located in Nusaybin district (Mardin, Türkiye; Elevation: 450 m, Latitude: 37.088586° N, Longitude: 41.223545° E). The walnut samples consisted of fallen fruits beneath the trees that were unsuitable for consumption and originated from an orchard where no plant protection products had been applied. Following collection, the samples were dried in the shade.

### 4.2. Pyrolysis System and Pyrolysis Liquids (PLs)

PLs were produced from agricultural walnut residue using a fixed-bed pyrolysis reactor equipped with a dual-cooling condensation system. The reactor temperature was controlled by a PID system, and the process was conducted under an inert atmosphere. Samples were heated at 5 °C/min to the target temperature ranges of 200–300 °C (W-1), 300–400 °C (W-2), 400–500 °C (W-3), and 500–600 °C (W-4). After reaching the desired temperature range, the process was maintained for approximately 2 h to complete thermal decomposition and condensable vapor recovery. The resulting condensates were collected as pyrolysis liquids and stored under appropriate conditions until analysis.

### 4.3. Determination of Chemical Profiles of PLs

#### 4.3.1. Total Phenolic Content (TPC)

The total phenolic content (TPC) of PLs was determined colorimetrically using the modified Folin–Ciocalteu method. Briefly, 100 µL of sample, 900 µL of distilled water, and 5 mL of 0.2 N Folin–Ciocalteu reagents were mixed sequentially. After allowing the mixture to stand for 8 min, 4 mL of Na_2_CO_3_ solution was added, vortexed, and then incubated in the dark for 2 h. Finally, UV absorbance values of the mixture were measured at 765 nm, and the results were evaluated by comparing them with the absorbance values of gallic acid standards at different concentrations [76,77,78].

#### 4.3.2. Total Flavonoid Content

The total flavonoid contents of PLs were calculated in terms of quercetin equivalents. For this purpose, 1.25 mL of distilled water (DH_2_O) and 75 µL of 5% NaNO_2_ solution were added to 0.25 mL of sample and allowed to stand for 6 min. Subsequently, 150 µL of 10% AlCl_3_·6H_2_O solution was added and incubated for 5 min. In the final step, 0.5 mL of 1 M NaOH was added, the volume was completed with 2.5 mL of distilled water, and measurements were performed at 510 nm wavelength using a UV-Vis spectrophotometer [79,80].

#### 4.3.3. Determination of Volatile Aromatic Compounds by GC-MS (SPME)

For the determination of aroma components in PLs, a Shimadzu GC-2010 Plus gas chromatograph coupled with a Shimadzu GCMS-QP2020 mass spectrometer (Shimadzu, Kyoto, Japan) was utilized. Separation was performed on a DB-HeavyWax column (60 m × 0.25 mm × 0.25 µm). The injection temperature was set at 250 °C, and the initial oven temperature was 40 °C. The temperature was increased to 80 °C at a rate of 3 °C/min, held for 1 min, then raised to 240 °C at 5 °C/min and maintained for 6 min. Helium (He) was used as the carrier gas with a flow rate of 1.05 mL/min. Sample aliquots of 2 mL were transferred into 20 mL vials, and after placing the fiber in the vial at 45 °C, adsorption was allowed in the headspace for 50 min. The fiber used was 50/30 µm × 2 cm divinylben-zene/carboxen/polydimethylsiloxane (DVB/CAR/PDMS, Supelco Inc., Bellefonte, PA, USA) type [81,82,83].

#### 4.3.4. Determination of Phenolic Profile by HPLC

PLs were first filtered through a 0.45 µm PVDF filter, and then 20 µL was injected into the C-18 column (5 µm, 4.6 × 250 mm, GL Sciences, Tokyo, Japan) of a Waters Alliance E2695 HPLC system to identify minor components. The mobile phase, prepared from methanol/acetonitrile (solvent B, 90:10 *v*/*v*) and ultrapure water (solvent A, pH = 2, with phosphoric acid), was used at a flow rate of 0.8 mL/min. The instrument was operated at 40 °C with an elution gradient consisting of 95–5% A-B for 2 min, 75–25% for 8 min, 60–40% for 10 min, 50–50% for 16 min, and 0–100% for 14 min. The system was held constant for 10 min and returned to initial conditions in 13 min. For quantification, standards of *p*-coumaric acid, caffeic acid, vanillic acid, and gallic acid were determined at 280 nm, and calibration curves were constructed. Finally, detection was performed using a PDA detector (Photodiode Array Detector-Waters 2996, Milford, MA, USA) and evaluated using Empower 3 instrument’s software (version e2695, Waters, Milford, MA, USA) [84,85,86].

#### 4.3.5. Elemental Composition of PLs

Elemental analysis of the liquid pyrolysis samples was performed using a PerkinElmer Optima 5300 DV ICP-OES instrument (PerkinElmer, Waltham, MA, USA). Since the samples were in liquid form, drying and grinding procedures were not applied. A specific volume from each sample was transferred to digestion tubes, and 5 mL of concentrated nitric acid (HNO_3_) was added. The samples were gently shaken to ensure complete contact with the acid and left overnight at room temperature. Subsequently, the digestion tubes were heated in a block digestion unit at 125 °C for 60 min. After cooling, 3 mL of 30% hydrogen peroxide (H_2_O_2_) was added to each tube, and digestion was continued at the same temperature. Additional 3 mL of H_2_O_2_ was added as needed until the solution became colorless. Additional nitric acid was added to prevent reduction in solution volume. After completion of digestion, the tubes were cooled to room temperature, and their contents were transferred to 50 mL volumetric flasks with 10% HCl or HNO_3_ solution and brought to final volume with 10% acid. To prevent silicon precipitation, the solutions were allowed to stand for 5–6 h, with filtration or centrifugation applied if necessary. Analyses were conducted under conditions of 1300 W RF power, 15 L/min plasma flow, 2.0 L/min auxiliary flow, 0.8 L/min nebulizer flow, and 1.5 mL/min sample uptake rate; two-point background correction was applied for each element, and measurements were performed in triplicate [87].

### 4.4. Determination of Antimicrobial Activity

#### 4.4.1. Determination of Antibacterial Activity

The antimicrobial activity of PLs against plant pathogenic bacteria (*C. michiganensis* subsp. *michiganensis*, *P. syringae* pv. *syringae*, *E. amylovora*, and *X. euvesicatoria*) was determined using the Agar Well Diffusion Method as described by Balouiri et al. [88]. Accordingly, the turbidity of 24 h bacterial cultures was adjusted to 0.5 McFarland standard [89]. Aliquots of 100 µL from these cultures were spread onto the surface of Mueller-Hinton Agar (Merck, Darmstadt, Germany), and 10 mm diameter wells were created using a cork borer. Stock PL solutions as well as 10% and 20% (*v*/*v*) diluted solutions were applied to the wells at 100 µL. Ciprofloxacin (5 µg, Oxoid, Basingstoke, UK) was used as a positive control, while 100 µL of sterile Tryptic Soy Broth served as the negative control. Plates were incubated at 35 °C for 24 h, after which inhibition zone diameters were measured in millimeters. All antibacterial assays were performed in three independent replicates (*n* = 3) using independent bacterial cultures. Initial screening assays were qualitative in nature, whereas confirmatory assays were subjected to statistical analysis. In addition to the screening assays conducted with diluted PLs (10% and 20%, *v*/*v*), confirmatory antibacterial evaluations were carried out using stock PLs (100% *v*/*v*) following the same protocol. Inhibition zone diameters were expressed as mean ± standard deviation. Statistical differences among treatments were evaluated using one-way analysis of variance (ANOVA), followed by Tukey’s HSD post hoc test, with significance accepted at *p* < 0.05. Furthermore, minimum inhibitory concentration (MIC) assays were conducted in triplicate for all four phytopathogenic bacteria, and MIC values were defined as the lowest concentration (µL/mL) of PL resulting in complete visible growth inhibition.

#### 4.4.2. Determination of Minimum Inhibitory Concentration (MIC)

The minimum inhibitory concentration (MIC) of walnut residue–derived PLs against phytopathogenic bacteria was determined according to the broth dilution method described by Balouiri et al. (2016) [88]. For this purpose, a series of six sterile test tubes was prepared. Each tube contained 2 mL of Mueller–Hinton Broth (MHB). The initial concentration of the pyrolysis liquid in the first tube was adjusted to 50 µL/mL, and subsequent tubes were prepared by two-fold serial dilution. Subsequently, 100 µL of bacterial suspensions standardized to 0.5 McFarland turbidity were inoculated into each tube [89]. The inoculated tubes were incubated at 35 °C for 24 h. Following incubation, bacterial growth was visually assessed based on turbidity. The MIC value was defined as the lowest concentration of pyrolysis liquid at which no visible bacterial growth was observed. All MIC assays were performed in triplicate to ensure reproducibility. Based on the agar well diffusion results, the W-4 fraction (500–600 °C), which exhibited the strongest antibacterial activity, was selected for MIC determination.

#### 4.4.3. Determination of Antifungal Activity

The antifungal activities of PLs were determined using the Poisoned Plate Method as described by Balouiri et al. [88]. In this context, Malt Extract Agar (Merck, Darmstadt, Germany) (MEA) plates containing 1%, 3%, 5%, 10%, and 20% (*v*/*v*) PLs were prepared. Sections of 10 mm diameter were taken from *F. oxysporum* and *V. dahliae* cultures and transferred to Petri plates. Additionally, as a positive control, sections from these fungal cultures were placed on MEA plates containing 50 µL cycloheximide (35 mg/mL stock solution). Cycloheximide was used as the positive control, whereas MEA plates without PL or cycloheximide served as the negative control. Following inoculation, Petri plates were incubated at 27 °C for 7 days. The experiment was conducted in a randomized complete block design with three replications. Following incubation, colony diameters were measured and recorded.

## 5. Conclusions

Based on our results, the systematic investigation of temperature-dependent pyrolysis of walnut agricultural residues demonstrated that thermal processing conditions exert a profound influence on the chemical composition and antimicrobial efficacy of resultant pyrolysis liquids. The GC–MS analysis revealed distinct temperature-dependent chemical profiles across four temperature ranges, with progressive modifications in compound distribution patterns and chemical class compositions. Progressive increases in pyrolysis temperature yielded substantial enhancements in total phenolic content, total flavonoid content, and volatile compound diversity, with the highest temperature fraction consistently exhibiting superior chemical complexity and bioactive potential. The antimicrobial screening revealed divergent activity patterns between bacterial and fungal pathogens. While antibacterial effects remained modest and pathogen-specific, antifungal activity proved remarkably robust and consistent. Complete mycelial growth inhibition of *F. oxysporum* and *V. dahliae* occurred at progressively lower concentrations with increasing pyrolysis temperature, directly correlating with enhanced phenolic content. Chemical characterization revealed complex mixtures dominated by acetic acid, 2-furancarboxaldehyde, and substituted phenols, with substantial contributions from carboxylic acids and aromatic compounds. Furfural decreased progressively with temperature, while propanoic acid increased, becoming dominant in the highest fraction. Nitrogen-containing compounds showed variable behaviors, with amino acids concentrated at intermediate temperatures and hydrazine derivatives exhibiting alternating patterns. Carboxylic acid content increased progressively, reflecting intensified thermal cracking. The highest temperature sample exhibited the greatest compositional diversity with unique lactones, heterocyclic structures, and aromatic. The complete absence of toxic heavy metals, coupled with beneficial mineral elements, confirms the safety profile for agricultural applications. These findings have immediate practical implications for sustainable agriculture and circular economy implementation. The transformation of walnut residues into bioactive pyrolysis liquids addresses agricultural residue valorization and renewable antimicrobial agent development. The demonstrated antifungal efficacy positions these liquids as promising bio-based plant protection products. Future research should prioritize formulation optimization, structure-activity relationship investigations, field efficacy trials, environmental fate assessments, and economic feasibility analyses.

## Figures and Tables

**Table 1:** Total phenolic and flavonoid content results of PLs.

Sample	Total Phenolic Content ^b^	Total Flavonoid Content ^c^
W-1	65.53 ± 8.00 ^a^	16.37 ± 1.89
W-2	146.05 ± 5.50	32.48 ± 1.41
W-3	233.62 ± 6.00	55.90 ± 3.77
W-4	321.18 ± 10.50	77.11 ± 2.36

^a^: Values are given as mean and standard deviation of 3 parallel measurements (W-1: PL produced at 200–300 °C, W-2: 300–400 °C, W-3: 400–500 °C, W-4: 500–600 °C). ^b^: Gallic acid equivalent phenolic content (Y = 0.8234x − 0.0053, R^2^ = 0.9953). ^c^: Quercetin equivalent flavonoid content (Y = 2.2059x − 0.0408, R^2^ = 0.9931).

**Table 2:** Comparative GC–MS profile of major compounds (>1% relative area) detected in W-1–W-4 samples.

Compound	200–300 °C [W-1] (%)	300–400 °C [W-2] (%)	400–500 °C [W-3] (%)	500–600 °C [W-4] (%)
Furfural	14.78	7.48	5.56	1.36
Propanoic acid	4.08	3.65	4.04	11.58
Acetic acid	6.39	ND	ND	ND
Formic acid	ND	2.21	2.70	3.83
Butyric acid	1.00	ND	ND	2.41
Propanedioic acid	7.01	ND	ND	ND
Hydrazine, 1,1-dimethyl-	12.18	ND	12.20	ND
Hydrazine, methyl-	ND	4.76	ND	ND
Methane, trinitro-	9.55	ND	ND	ND
Nitramide	7.98	ND	ND	ND
L-Alanine	3.11	14.98	ND	ND
1,2-Propanediamine	4.25	9.74	ND	ND
1,2,3-Propanetriol	ND	3.88	ND	ND
γ-Butyrolactone	1.94	2.68	ND	ND
Guaiacol	1.54	ND	ND	3.04
Oxybenzene	1.58	1.21	1.36	ND
Pyridine	1.02	1.27	1.43	3.63
2(3H)-Furanone, dihydro-	ND	ND	ND	8.86
9,12-Octadecadienoic acid (Z,Z)-	1.75	ND	ND	ND
9-Octadecenoic acid, (E)-	1.81	ND	ND	ND
Propane, 1-fluoro-	5.30	ND	ND	ND
1,2-diethyldiborane-D10	ND	3.43	9.41	1.98
1-propanol-O-D	ND	6.49	ND	ND
2-propanol	ND	5.04	ND	ND
Acetamide	ND	1.48	1.46	4.43
Butanoic acid	ND	4.34	ND	ND
N,N-Dimethylacetamide	ND	7.04	ND	ND
1,4-Dithiane	ND	ND	4.95	ND
1-Propanol, 2-amino-	ND	ND	10.64	ND
Butanoic acid, 4-chloro-	ND	ND	2.95	ND
Hydrazine, methyl-, oxalate	ND	ND	3.17	ND
Methanamine, N-methoxy-	ND	ND	6.81	ND
Methane, nitro-	ND	ND	7.73	ND
N-Ethyl-N-methyl-formamide	ND	ND	4.31	ND
N-methyl-N-(methyl-d3) aminoheptane	ND	ND	1.19	ND
o-Ethylhydroxylamine	ND	ND	2.34	ND
1,2-Ethanediamine, N-(2-aminoethyl)-	ND	ND	ND	1.21
2(3H)-Furanone, dihydro-5-methyl-	ND	ND	ND	1.09
2,5-Hexanedione	ND	ND	ND	1.05
2,5-Pyrrolidinedione, 1-methyl-	ND	ND	ND	1.27
2-Amino-5,6-dihydro-4,4,6-trimethyl-4H-1,3-oxazine	ND	ND	ND	1.23
2H-Pyran-2-one, tetrahydro-	ND	ND	ND	1.65
2-Propanone, 1-(acetyloxy)-	ND	ND	ND	1.16
Benzenesulfonic acid, 4-hydroxy-	ND	ND	ND	4.01
Pentanoic acid, 4-oxo-, methyl ester	ND	ND	ND	1.17
Propane, 2-methoxy-2-propoxy-	ND	ND	ND	1.77
Pyridine, 3-methoxy-	ND	ND	ND	1.18
Tetrahydrofurfuryl alcohol	ND	ND	ND	1.47

ND: not detected.

**Table 3:** Comparative GC–MS profile grouped by chemical class (≥1% relative area).

Chemical Class	Compound	200–300 °C [W-1] (%)	300–400 °C [W-2] (%)	400–500 °C [W-3] (%)	500–600 °C [W-4] (%)
Organic acids & fatty acids	Acetic acid	6.39	ND	ND	ND
	Formic acid	ND	2.21	2.70	3.83
	Propanoic acid	4.08	3.65	4.04	11.58
	Butyric acid	1.00	ND	ND	2.41
	Butanoic acid	ND	4.34	ND	ND
	Butanoic acid, 4-chloro-	ND	ND	2.95	ND
	Propanedioic acid	7.01	ND	ND	ND
	Pentanoic acid, 4-oxo-, methyl ester	ND	ND	ND	1.17
	9,12-Octadecadienoic acid (Z,Z)	1.75	ND	ND	ND
	9-Octadecenoic acid (E)	1.81	ND	ND	ND
Furans & lactones	Furfural	14.78	7.48	5.56	1.36
	γ-Butyrolactone	1.94	2.68	ND	ND
	2(3H)-Furanone, dihydro-	ND	ND	ND	8.86
	2(3H)-Furanone, dihydro-5-methyl-	ND	ND	ND	1.09
	2H-Pyran-2-one, tetrahydro-	ND	ND	ND	1.65
Phenolics & aromatic compounds	Guaiacol	1.54	ND	ND	3.04
	Oxybenzene	1.58	1.21	1.36	ND
	Benzenesulfonic acid, 4-hydroxy-	ND	ND	ND	4.01
	Pyridine	1.02	1.27	1.43	3.63
	Pyridine, 3-methoxy-	ND	ND	ND	1.18
Alcohols & polyols	1,2,3-Propanetriol	ND	3.88	ND	ND
	1-Propanol-O-D	ND	6.49	ND	ND
	2-Propanol	ND	5.04	ND	ND
	1-Propanol, 2-amino-	ND	ND	10.64	ND
	Tetrahydrofurfuryl alcohol	ND	ND	ND	1.47
Nitrogen-containing compounds	Hydrazine, 1,1-dimethyl-	12.18	ND	12.20	ND
	Hydrazine, methyl-	ND	4.76	ND	ND
	Hydrazine, methyl-, oxalate	ND	ND	3.17	ND
	Nitramide	7.98	ND	ND	ND
	Methane, trinitro-	9.55	ND	ND	ND
	Methane, nitro-	ND	ND	7.73	ND
	Methanamine, N-methoxy-	ND	ND	6.81	ND
	N,N-Dimethylacetamide	ND	7.04	ND	ND
	N-Ethyl-N-methyl-formamide	ND	ND	4.31	ND
	Acetamide	ND	1.48	1.46	4.43
	L-Alanine	3.11	14.98	ND	ND
	1,2-Propanediamine	4.25	9.74	ND	ND
	1,2-Ethanediamine, N-(2-aminoethyl)-	ND	ND	ND	1.21
	o-Ethylhydroxylamine	ND	ND	2.34	ND
Other compounds	Propane, 1-fluoro-	5.30	ND	ND	ND
	1,2-Diethyldiborane-D10	ND	3.43	9.41	1.98
	1,4-Dithiane	ND	ND	4.95	ND
	2,5-Hexanedione	ND	ND	ND	1.05
	2,5-Pyrrolidinedione, 1-methyl-	ND	ND	ND	1.27
	2-Amino-5,6-dihydro-4,4,6-trimethyl-4H-1,3-oxazine	ND	ND	ND	1.23
	2-Propanone, 1-(acetyloxy)-	ND	ND	ND	1.16
	Propane, 2-methoxy-2-propoxy-	ND	ND	ND	1.77

**Table 4:** Phenolic profile of PLs determined by HPLC.

Sample (mg/L)	Gallic Acid	Caffeic Acid	Vanillic Acid	*p*-Coumaric Acid
W-1	9.785	0.225	1.445	0.105
W-2	8.900	0.755	1.180	0.415
W-3	8.240	1.280	2.315	0.505
W-4	8.810	1.525	1.620	0.600

**Table 5:** Elemental analysis of PLs by ICP-OES (mg/kg).

Samples	Cu	Zn	Fe	Mn	Mg	Sn	K	Ca	Na	Cd	Ni	Pb	Hg	Cr
W-1	<0.05	451.69	315.60	<0.01	283.50	<0.05	250	3750	750	<0.05	<0.05	<0.05	<0.05	<0.05
W-2	<0.05	452.75	1287.50	<0.01	147.10	<0.05	250	10,875	875	<0.05	<0.05	<0.05	<0.05	<0.05
W-3	<0.05	425.49	1077.50	<0.01	306.70	<0.05	125	3875	750	<0.05	<0.05	<0.05	<0.05	<0.05
W-4	<0.05	650.06	1199.00	<0.01	358.80	<0.05	250	3875	750	<0.05	<0.05	<0.05	<0.05	<0.05

**Table 6:** Confirmatory antibacterial screening of stock pyrolysis liquids (100% *v*/*v*) against phytopathogenic bacteria.

Plant Pathogens	W-1 (mm)	W-2 (mm)	W-3 (mm)	W-4 (mm)	Positive Control (mm)	Negative Control (mm)
*C. michiganensis*	20.0 ± 1.0 ^a^	23.0 ± 1.5 ^b^	25.0 ± 1.0 ^c^	32.0 ± 2.0 ^d^	25.0 ± 1.0 ^c^	0
*E. amylovora*	23.0 ± 1.5 ^a^	25.0 ± 1.0 ^b^	30.0 ± 2.0 ^c^	38.0 ± 2.5 ^d^	25.0 ± 1.0 ^b^	0
*P. syringae*	27.0 ± 2.0 ^a^	30.0 ± 1.5 ^b^	35.0 ± 2.0 ^c^	45.0 ± 3.0 ^d^	25.0 ± 1.0 ^a^	0
*X. euvesicatoria*	22.0 ± 1.0 ^a^	25.0 ± 1.5 ^b^	30.0 ± 2.0 ^c^	35.0 ± 2.5 ^d^	23.0 ± 1.0 ^a^	0

Note: Values are expressed as mean ± standard deviation of three independent replicates (*n* = 3). Different letters within the same row indicate statistically significant differences among treatments (one-way ANOVA followed by Tukey’s HSD test, *p* < 0.05).

**Table 7:** Antibacterial activity of pyrolysis liquids against phytopathogenic bacteria at 10% and 20% (*v*/*v*) concentrations determined by agar well diffusion method.

Applications	Plant Pathogens	W-1 (*v*/*v*) Avg. (mm)	W-2 (*v*/*v*) Avg. (mm)	W-3 (*v*/*v*) Avg. (mm)	W-4 (*v*/*v*) Avg. (mm)	N. Control Avg. (mm)	P. Control Avg. (mm)
10% PL (*v*/*v*)	*C. michiganensis*	0	0	0	0	0	25
	*Erwinia amylovora*	0	0	0	0	0	25
	*Pseudomonas syringae* pv. *syringae*	0	0	0	0	0	30
	*Xanthomonas euvesicatoria*	0	0	0	0	0	20
20% PL (*v*/*v*)	*C. michiganensis*	0	0	0	0	0	35
	*Erwinia amylovora*	0	0	0	15	0	25
	*Pseudomonas syringae* pv. *syringae*	0	0	0	0	0	30
	*Xanthomonas euvesicatoria*	0	0	0	0	0	10

**Table 8:** Minimum inhibitory concentration (MIC) (µL/mL) values of the highest-temperature pyrolysis liquid fraction (W-4; 500–600 °C) against phytopathogenic bacteria.

Phytopathogenic Bacteria	MIC (µL/ mL)
*Clavibacter michiganensis* subsp. *michiganensis*	12.50
*Xanthomonas euvesicatoria*	12.50
*Pseudomonas syringae* pv. *syringae*	12.50
*Erwinia amylovora*	25.00

Note: Values represent the MIC determined from three independent replicates.

**Table 9:** Minimum fully inhibitory concentrations of pyrolysis liquids against fungal pathogens.

Pathogen	W-1	W-2	W-3	W-4
*F. oxysporum*	20%	10%	5%	5%
*V. dahliae*	20%	10%	5%	5%

Values indicate the lowest concentration resulting in complete mycelial growth inhibition under poisoned plate assay conditions.

## Data Availability

The data that support the findings of this study are available from the corresponding authors upon reasonable request.
